# A five-DNA methylation signature act as a novel prognostic biomarker in patients with ovarian serous cystadenocarcinoma

**DOI:** 10.1186/s13148-018-0574-0

**Published:** 2018-11-16

**Authors:** Wenna Guo, Liucun Zhu, Minghao Yu, Rui Zhu, Qihan Chen, Qiang Wang

**Affiliations:** 1 State Key Laboratory of Pharmaceutical Biotechnology, School of Life Sciences, Nanjing University, Nanjing, China; 2 School of Life Sciences, Shanghai University, Shanghai, China

**Keywords:** Biomarker, DNA methylation, OSC, Prognosis, Risk stratification

## Abstract

**Background:**

Ovarian cancer is the most fatal tumor of the female reproductive system and the fifth leading cause of cancer death among women in the USA. The prognosis is poor due to the lack of biomarkers for treatment options.

**Results:**

The methylation array data of 551 patients with ovarian serous cystadenocarcinoma (OSC) in The Cancer Genome Atlas (TCGA) database were assessed in this study to explore the methylation biomarkers associated with prognosis and improve the prognosis of patients. These patients were divided into training (first two thirds) and validation datasets (remaining one third). A five-DNA methylation signature was found to be significantly associated with the overall survival of patients with OSC using the Cox regression analysis in the training dataset. The Kaplan–Meier analysis showed that the five-DNA methylation signature could significantly distinguish the high- and low-risk patients in both training and validation sets. The receiver operating characteristic (ROC) analysis further confirmed that the five-DNA methylation signature exhibited high sensitivity and specificity to predict the prognostic survival of patients. Also, the five-DNA methylation signature was not only applicable in patients of different ages, stages, histologic grade, and size of residual tumor after surgery but also more accurate in predicting OSC prognosis compared with known biomarkers.

**Conclusions:**

This five-DNA methylation signature demonstrated the potential of being a novel independent prognostic indicator and served as an important tool for guiding the clinical treatment of OSC to improve outcome prediction and management for patients. Hence, the findings of this study might have potential clinical significance.

**Electronic supplementary material:**

The online version of this article (10.1186/s13148-018-0574-0) contains supplementary material, which is available to authorized users.

## Background

Ovarian cancer is the most lethal cancer of the female reproductive system and the fifth leading cause of cancer death among women in the USA with an estimated 22,240 new cases and 14,070 deaths expected to occur in 2018 [1, 2]. Ovarian serous cystadenocarcinoma (OSC), a common type of ovarian cancer, accounts for about 90% of all ovarian cancers [2]. The standard treatment consists of cytoreductive surgery followed by a combination of platinum- and taxane-based chemotherapy [3]. Although advances in treatment technology in the last few decades have substantially improved the average survival time, the cure rates remain relatively unchanged [4]. The overall 5-year survival probability of women diagnosed with ovarian cancer is still less than 50% (47%) [1]. Assessment of patients prior to therapy might enable a risk-adapted approach and hence offer an opportunity to provide improved personalized treatment. Physicians can direct low-risk patients to conventional treatments, while high-risk patients can be channeled to trials of novel therapies. This selection may enhance the ability of clinical trials to demonstrate clinical benefits. Therefore, determining high-risk patients with OSC and improving the clinical outcome are urgently needed for current clinical management. The identification of highly specific, sensitive, and independent predictive prognostic biomarkers that will allow the stratification of care is essential.

DNA methylation is well known to be associated with ovarian cancer and has great potential to serve as a biomarker in screening the disease, monitoring response to therapy, and predicting the prognosis [5, 6]. The methylation of particular subsets of CpG islands may have consequences for specific processes of tumorigenesis [7]. Aberrant DNA methylation occurs commonly in tumors and is recognized as one of the earliest distinguishing molecular characteristic in carcinogenesis [8, 9]. A number of genes have been identified as being hypermethylated or silenced in ovarian cancer [10]. Thus, cancer methylation studies hold great promise in revealing potential biomarkers for improving the survival rate. Using DNA methylation as a biomarker has several advantages over other molecular markers, including the relative stability of DNA methylation both in vivo and ex vivo [11]; need for a smaller amount of tissues to obtain adequate DNA for methylation analysis [12]; and relative accuracy thanks to quantitative assay because DNA methylation measurements can be compared with absolute reference points [13]. An increasing number of reports are available about the potential of DNA methylation as a prognostic biomarker [14]. For instance, patients with higher methylation levels of *ABCA1* have shorter overall survival [10]; hypomethylation of CpG sites within the *MSX1* gene is associated with resistant high-grade serous ovarian cancer [15]; and *OPCML* gene promoter methylation can serve as a useful biomarker for predicting the prognosis of patients with ovarian cancer [5]. However, the use of genome-wide methylation analysis in clinical practice is limited by the large sets of DNA methylation identified and the difficulties in complex statistical analyses. Moreover, the reproducibility of prognostic methylation signature identified is limited by different specimens and the lack of adjustment for major confounding factors [16].

Consequently, the whole-genome methylation profiles of tumor tissues from patients with OSC in The Cancer Genome Atlas (TCGA) database were analyzed in this study to identify DNA methylation biomarkers so as to explore the utility of DNA methylation analysis for cancer prognosis. The potential clinical significance of methylation biomarkers serving as molecular prognostic markers was examined using Kaplan–Meier method and receiver operating characteristic (ROC) analysis. Furthermore, the independence and reproducibility of identified methylation biomarkers in different groups were also investigated.

## Results

### Clinical characteristics of the patients

All 551 patients in this study were clinically and pathologically diagnosed with OSC. The median age and median survival of these patients were 60 years (range, 30–89 years) and 1227 days, respectively. The 3-year overall survival (OS) rate of all patients was 51.60%. The clinical stage was defined according to the Federation Internationale des Gynaecologistes et Obstetristes (FIGO) staging system. The tumor histologic grade was assigned according to the World Health Organization criteria. OSC was divided into stages I, II, III, and IV, and the neoplasm histologic grade included G2, G3, and G4. Anatomic neoplasm subdivisions were obtained from different positions, including left, right, and bilateral. Tumor residual diseases were dichotomized into no macroscopic disease, 1–10 mm, 11–20 mm, and > 20 mm. The clinicopathological characteristics of patients are summarized in Table 1.

**Table 1:** Clinicopathological characteristics of OSC patients from TCGA

Characteristics	Groups	Patients					
		Total (*N* = 551)		Training dataset (*N* = 368)		Validation dataset (*N* = 183)	
		No.	%	No.	%	No.	%
Age at diagnosis	Median	59		60		59	
	Range	26–89		34–87		30–89	
	< 60	286	50.44	187	50.95	104	63.41
	≥ 60	265	46.74	182	49.59	79	48.17
FIGO stage	I	15	2.65	5	1.36	10	6.10
	II	27	4.76	12	3.27	15	9.15
	III	423	74.60	287	78.20	136	82.93
	IV	82	14.46	62	16.89	20	12.20
	Unknown	4	0.71	2	0.54	2	1.22
Histologic grade*	G2	69	12.17	25	6.81	44	26.83
	G3	478	84.30	341	92.92	137	83.54
	G4	1	0.18	1	0.27	0	0.00
	Others	3	0.53	1	0.27	2	1.22
Tumor residual (mm)	No macroscopic disease	116	20.46	67	18.26	49	29.88
	1–10	244	43.03	191	52.04	53	32.32
	11–20	33	5.82	16	4.36	17	10.37
	> 20	105	18.52	64	17.44	41	25.00
	Unknown	53	9.35	33	8.99	25	15.24
Anatomic subdivision	Bilateral	383	67.55	253	68.94	130	79.27
	Left	78	13.76	55	14.99	23	14.02
	Right	62	10.93	40	10.90	22	13.41
	Unknown	28	4.94	20	5.45	8	4.88

*G1 and GB/GX were excluded in this study as these tumors may have a different biological behavior

### Identification of DNA methylation markers associated with the OS of patients in the training dataset

The univariate Cox proportional hazard regression analysis (see the “Materials and methods” section) was performed using the methylation levels as variables in the training dataset to identify DNA methylation markers associated with the OS of patients with OSC. As a result, a total of 1630 DNA methylation sites were found to be significantly associated with the OS of patients (*P* < 0.05). Subsequently, multivariate Cox regression, stepwise regression, and screening were performed for these 1282 DNA methylation sites, and a hazard ratio model consisting of 5 methylation sites (cg05254747, cg13652336, cg25123470, cg06038133, and cg04907664) was identified as the optimum prognostic model for predicting the OS of patients. In this model, these 5 methylation sites were all significantly (*P* < 0.05) associated with the OS of patients. The risk scoring formula of these 5 methylation sites was obtained: Risk score = − 1.034 × *β* value of cg05254747 + 2.433 × *β* value of cg13652336 + 1.552 × *β* value of cg25123470 + 2.284 × *β* value of cg06038133–1.030 × *β* value of cg04907664. Obviously, the hypermethylation levels of cg13652336, cg25123470, and cg06038133 were associated with a higher risk, whereas the hypomethylation levels of cg05254747 and cg04907664 were associated with a higher risk. The corresponding gene symbol of these 5 sites was *SLC39A14*, *PREX2*, *KCNIP2*, *CORO6*, and *EFNB1*, respectively. The chromosomal locations of these 5 methylation sites and related log-rank test *P* values are shown in Additional file 1: Table S1.

### Association between five-DNA methylation signature and patient OS in the training and validation datasets

**Fig. 1:**
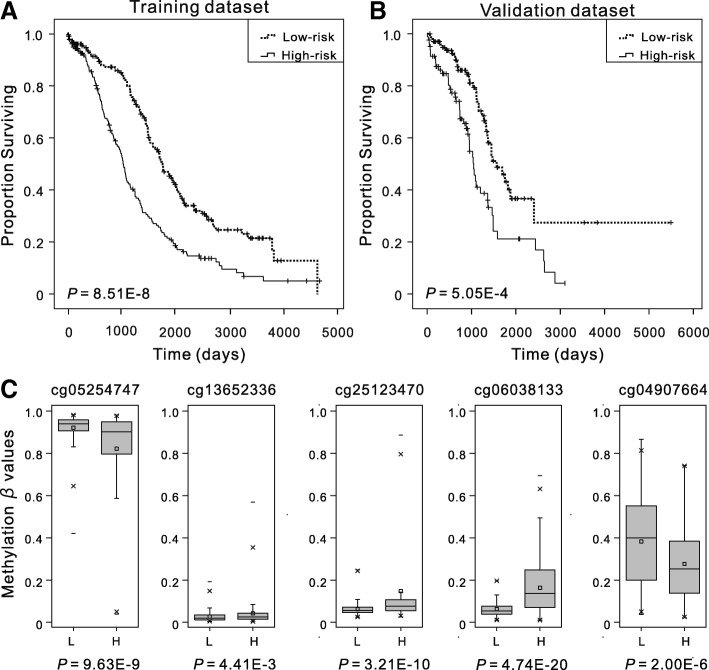
Overall survival (OS) and methylation levels of different patient cohorts. The Kaplan–Meier estimates of the OS for high-risk and low-risk patient cohorts grouping by the five-DNA methylation signature in the training dataset (*N* = 368) (**a**) and the validation dataset (*N* = 183) (**b**). The OS differences between the two groups were determined by the two-sided log-rank test. It can be concluded that higher risk scores are significantly associated with worse OS (*P* < 0.001). **c** Boxplots of methylation *β* values in samples of patients in high-risk and low-risk groups in the training dataset. “L” and “H” refer to the low-risk and high-risk group, respectively. Mann–Whitney *U* test was used to determine the differences between the two groups, and *P* values are shown below the graphs

Hazard ratios (HRs) from the Cox regression analysis indicated that the five-DNA methylation signature was significantly associated with the OS of patients (*P* < 0.001, HR 2.72, 95% CI 2.03–3.65). The Kaplan–Meier analysis was performed in the training and validation datasets to determine the potential predictive value of this five-DNA methylation signature in the prognosis. The five-DNA methylation signature was assigned to each patient in the high-risk (*N* = 174) or the low-risk (*N* = 174) group in the training dataset using the median of prognostic risk scores as the cutoff point. The mean OS in the high-risk and low-risk groups was 1080 days and 1499 days, respectively. The patients in the high-risk group had a significantly (*P* < 0.001) worse prognosis (Fig. 1a). A similar result was observed in the validation dataset (Fig. 1b). These results showed that the novel five-DNA methylation signature could distinguish high-risk patients from low-risk patients, implying its significance in the prognostic prediction of OSC. Meanwhile, the individual methylation levels of these five methylation sites in patients in the high- and low-risk groups were analyzed. As a result, high-risk patients exhibited significantly lower methylation levels for cg05254747 and cg04907664 and significantly higher methylation levels for the other three methylation sites in both training (Fig. 1c) and validation datasets (Additional file 1: Figure S1) (*P* < 0.01, Mann–Whitney *U* test), which were consistent with the previous results.

### Evaluation of the predictive performance of the five-DNA methylation signature using ROC analysis

**Fig. 2:**
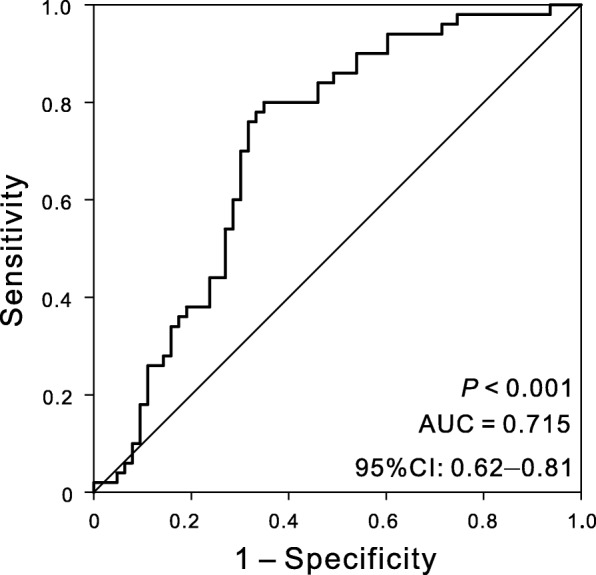
ROC analysis of sensitivity and specificity for the five-DNA methylation signature in predicting the OS of patients in the validation dataset. The AUC was 0.715 (95% CI = 0.62–0.81) (*P* < 0.001)

The sensitivity and specificity of the five-DNA methylation signature in predicting survival were evaluated using the ROC analysis to further assess the predictive accuracy of the five-DNA methylation signature in the validation dataset. The AUC of the five-DNA methylation signature was 0.715 (*P* < 0.001, 95% CI 0.62–0.81) (Fig. 2), indicating that the five-DNA methylation signature had high sensitivity and specificity. Therefore, it could be used to predict the prognostic survival of patients with OSC with high accuracy, and it might have potentially great significance in clinical application.

### Predictive performance of the five-DNA methylation signature based on different regrouping methods

**Fig. 3:**
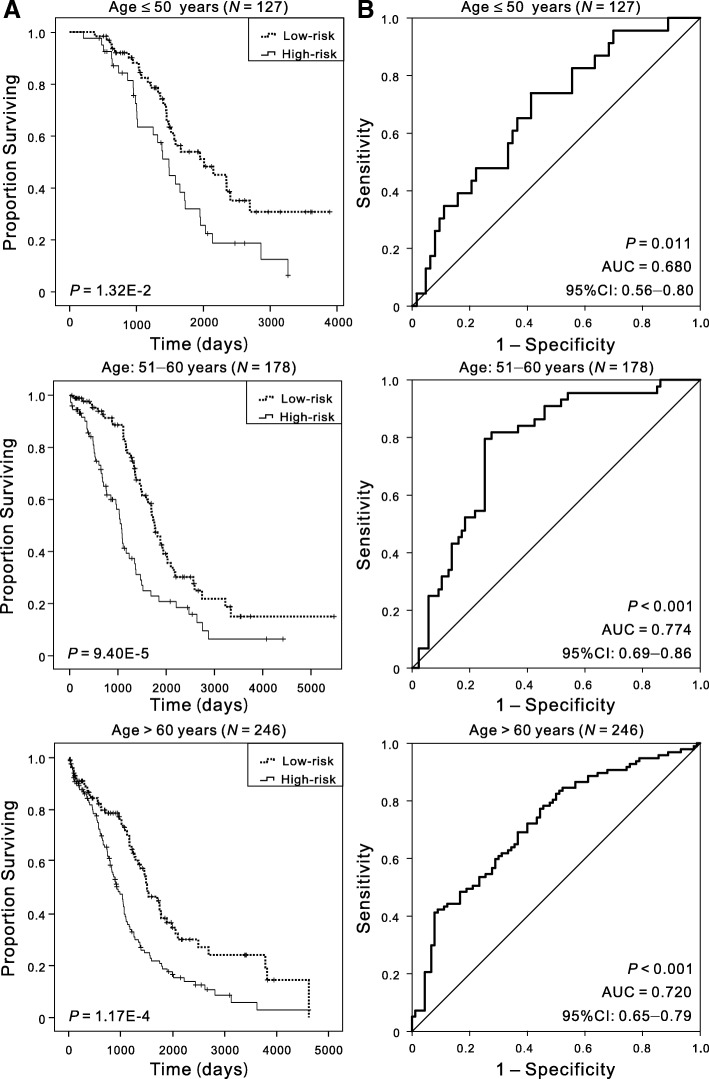
Kaplan–Meier and ROC analyses of patients with OSC in different age cohorts, grouping based on their ages at initial diagnosis: ≤ 50 (*N* = 127, 23.05%), 51–60 (*N* = 178, 32.30%), > 60 (*N* = 246, 44.65%). **a** Kaplan–Meier analysis with two-sided log-rank test was performed to estimate the differences in OS between the low-risk and high-risk patients. **b** ROC curves of the five-DNA methylation signature were used to demonstrate the sensitivity and specificity in predicting the OS of patients

Furthermore, several factors were associated with prognostic survival, including age [1, 17], stage [18], histologic grade [19], and size of residual tumor after cytoreductive surgery [20], and the reproducibility was poor in the prognostic markers identified by different groups [5]. Regrouping was carried out based on different clinicopathological characteristics so as to confirm that this five-DNA methylation signature was of high applicability and could precisely predict the OS of patients. Gillen et al. found that increasing age was correlated with shorter survival [17], and Chi et al. found that patient age might serve as a significant prognostic factor for ovarian carcinoma [21]. Patients were divided into three cohorts based on their ages at initial diagnosis: ≤ 50 (*N* = 127, 23.05%), 51–60 (*N* = 178, 32.30%), and > 60 (*N* = 246, 44.65%), to analyze the clinical effect of the five-DNA methylation signature in patients with different ages. Kaplan–Meier curves showed that patients in the low-risk group had significantly (*P* < 0.01) longer OS, and the AUC value was 0.680, 0.774, and 0.720 respectively for the three age cohorts (Fig. 3), suggesting that the five-DNA methylation signature was independent of age. Patients in stages III and IV had significantly shorter OS compared with patients in stages I and II [22], and the 5-year survival of women diagnosed with distant-stage disease was only 29% [1]. Despite the markedly different outcomes by the extent of disease, the OS was obviously different in high- and low-risk groups, and the AUC in stages I and II and stages III and IV cohorts was 0.778 and 0.735, respectively (Additional file 1: Figure S2). As for the histologic grade, considering the number of samples, we verified the predictive performance of the five-DNA methylation signature in G2 (*N* = 69) and G3 (*N* = 478). Irrespective of grades, the patients in the high-risk group had significantly (*P* < 0.05) shorter OS, and the AUC values were 0.696 and 0.740 (Additional file 1: Figure S3). The anatomic subdivisions from left alone and right alone were combined as unilateral cohorts for these analyses due to small numbers. The differences (*P* < 0.001) in the OS between the two groups were also observed, and the AUC values in all the subgroups were more than 0.72, in both unilateral (*N* = 140) and bilateral cohorts (*N* = 383) (Additional file 1: Figure S4). Recent investigations highlighted that the distribution of residual disease was an important predictor and a determinant of OS of patients [23]. The present data showed that the five-DNA methylation signature could provide a fairly better reference for different residual disease cohorts owing to the effectiveness of risk stratification (Additional file 1: Figure S5). All these results indicated that the signature showed satisfactory applicability when patients were regrouped by different clinicopathological characteristics, suggesting that the signature was an independent applicable prognostic predictor of patient survival. The results are summarized in Table 2.

**Table 2:** Results of Kaplan–Meier and ROC analysis based on different regrouping methods

Regrouping factors	Group	Sample size	Kaplan–Meier, *P* value	AUC	95% CI of AUC
Age at diagnosis	≤ 50	127	1.32E−02	0.680	0.56–0.80
	51–60	178	9.40E−05	0.774	0.69–0.86
	> 60	246	1.17E−04	0.720	0.65–0.79
FIGO stage	I and II	42	6.76E−02	0.778	0.59–0.96
	III and IV	505	1.03E−09	0.735	0.69–0.79
Histologic grade	G2	69	2.91E−02	0.696	0.53–0.86
	G3	478	3.26E−09	0.740	0.69–0.79
Anatomic subdivision	Unilateral	140	4.27E−04	0.753	0.66–0.85
	Bilateral	383	4.73E−07	0.727	0.67–0.79
Tumor residual disease (mm)	No macroscopic disease	116	1.17E−03	0.795	0.67–0.92
	1–10	255	1.16E−03	0.665	0.59–0.76
	> 10	138	5.18E−05	0.770	0.68–0.86

### Comparison of the five-DNA methylation signature with other known prognostic biomarkers

**Fig. 4:**
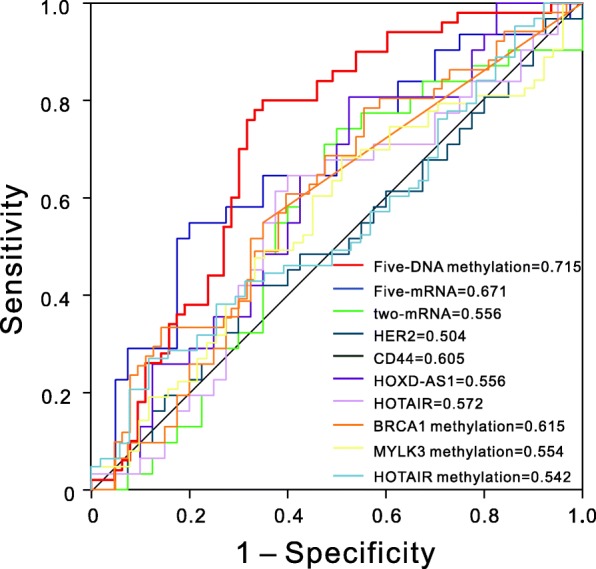
ROC curves show the sensitivity and specificity of the five-DNA methylation signature and other known biomarkers in predicting the OS of patients

In addition, several prognostic biomarkers were identified in previous studies. For instance, Luo et al. demonstrated that the expression of *HER2* was a predictor of poor prognosis for ovarian cancer [24]. The expression model of *MANF* combined with *DOCK11* was associated with the prognostic outcomes of patients with OSC, and the model could potentially serve as a novel prognostic indicator [25]. The methylation of the *BRCA1* promoter was associated with a poor patient outcome [26]. Expression of *HOTAIR* was an independent prognostic factor of OS, and its surrogate DNA methylation signature indicated carboplatin resistance in ovarian cancer [27, 28]. The sensitivity and specificity of known biomarkers from other studies were chosen to be evaluated in the validation dataset so as to verify whether the five-DNA methylation signature had the advantage of stable and reliable performance. The ROC analyses for other known biomarker is just as the analysis for our five-DNA methylation signature, and the results showed that the five-DNA methylation signature outperformed other known prognostic biomarkers, including the types of mRNA, lncRNA, and DNA methylation. And statistical comparison using *Z* test revealed that our signature had significantly higher (*P* < 0.05) predictive performance than most of the other known biomarker. The AUCs of these biomarkers are shown in Fig. 4 and Additional file 1: Table S2. All these results inspiringly revealed that the five-DNA methylation signature provided better stability and reliability in predicting the OS of patients with OSC and was a superior predictor. Additionally, the expression of the genes corresponding to the five DNA methylation sites and genes in the five-mRNA signature [29] whose accuracy is second only to the five-DNA methylation signature were also analyzed. And the results demonstrated that the latter genes had higher fold changes in the comparison of high- and low-risk patients, and no difference was noted in the expression of almost all the former five genes in this study (Additional file 1: Figure S6).

## Discussion

Molecular signatures have been proven to predict the clinical prognosis in different kinds of tumors [6, 10, 30, 31]. For instance, the methylation of *PCDH19* predicted a poor prognosis of hepatocellular carcinoma [31]; the methylation of *DFNA5* showed strong potential as a prognostic biomarker for breast cancer; and the signature of *CXCL11* combined with *HMGA2* could precisely predict the OS of patients with high-grade serous ovarian cancer [30]. However, many of these studies were limited by either small sample size or lack of validation of the biomarker as an independent prognostic biomarker. Some studies showed that combinations of DNA methylation as biomarkers achieved higher sensitivity and specificity compared with individual DNA methylation [12]. In the present study, a five-DNA methylation signature significantly associated with the OS of patients with OSC was predicted based on genome-wide DNA methylation analysis using the Cox regression and ROC analyses. The five-DNA methylation signature also performed well in differentiating low- and high-risk groups and in associated log-rank tests with significant *P* values, demonstrating that it was an independent predictor of patient survival when adjusted by age, FIGO stages, histologic grade, and residual disease after cytoreductive surgery. Furthermore, the results of the univariate Cox regression and Kaplan–Meier analyses for the five individual methylation sites were not as good as for the combination of these five-DNA methylation sites in both training and validation datasets, indicating that a combination of methylation sites might offer a better potential to fulfill much more sensitive and specific prognostic tests for patients with OSC.

Researchers have revealed that the aforementioned five methylation sites may be crucial in cancer development. *SLC39A14* has been identified as an independent factor for predicting the biochemical recurrence-free survival of patients with prostate cancer, and the decreased expression of *SLC39A14* is associated with the tumor aggressiveness of human prostate cancer [32]. *PREX2* (also known as *P-Rex2*) was highly expressed in the brain, heart, skeletal muscle, placenta, and lymph node and promoted cancer cell migration and/or invasion [33–35]. The *CORO6* promoter was frequently methylated in renal cell cancer [36]. The expression of *EFNB1* was related to the metastasis of breast cancer, and its enhanced expression conferred a poor prognosis [37]. The elevated co-expression of *NGFR*, *EFNB1*, and *APP* was associated with longer overall and metastasis-free survival of patients with breast cancer [38]. In addition, SLC39A14 participates in manganese ion transmembrane transporter activity; KCNIP2 regulates calcium ion binding; PREX2 and EFNB1 are involved in the G-protein coupled receptor signaling pathway and ephrin receptor signaling pathway, respectively. Although the functional mechanism of these five genes still needs further study, their methylation has significant correlations with the prognosis of patients with OSC and may serve as a potential therapeutic target for OSC.

Meanwhile, a comparison of the five-DNA methylation signature with other known prognostic biomarkers showed that it had distinctly higher sensitivity and specificity in the outcome prediction of OSC. The five-mRNA signature identified in a previous study [29] also had high accuracy surpassed only by the five-DNA methylation signature. However, these five mRNAs were completely different from the genes that corresponded to the five DNA methylation sites in this study. The expression analysis of these ten genes between high- and low-risk patients indicated that one possible reason was that mRNAs acting as biomarkers had larger fold changes in the expression level to be captured by our statistical methods. Further, it is generally believed that DNA methylation has an effect on the gene regulation with several exceptions [6, 39]. The potential association between the methylation level and the gene expression levels of these five genes demonstrated that only the expression of *SLC39A14* and *CORO6* was significantly (*P* < 0.05) correlated with the methylation levels at cg05254747 and cg06038133 sites, respectively, and no statistically significant association was observed between the methylation level and the expression of the other three genes. Further studies should be performed to establish a better prognostic biomarker for the combination of mRNAs and DNA methylation signature.

## Conclusion

In conclusion, using genome-wide analysis of DNA methylation data of 551 patients with OSC, this study showed that a five-DNA methylation signature was significantly associated with the OS of patients, and its practical value in patients with different ages, FIGO stages, histologic grades, and some other clinical features was confirmed. Therefore, the five-DNA methylation signature may potentially be used as a novel independent prognostic biomarker to predict the OS of patients with OSC. Further clinical studies on the functional mechanism of the five-DNA methylation signature should be examined for the possibility of its participation in the carcinogenesis.

## Materials and methods

### DNA methylation data in OSC tissues from TCGA dataset

The DNA methylation data of patients with OSC were downloaded from TCGA database [40]. TCGA level 3 methylation data and related clinical information for patients were obtained. TCGA DNA methylation data (level 3) were obtained using Infinium HumanMethylation27 BeadChip (Illumina Inc., CA, USA), and the genomic coordinates of the CpGs were based on GRCh38. All DNA methylation levels were expressed as *β* values, calculated as *M*/(*M* + *U*), where *M* is the signal from methylated beads, and *U* is the signal from unmethylated beads at the targeted CpG site. Only the data including patients with their clinical survival information were selected to analyze the correlation between DNA methylation levels and the corresponding survival in OSC. Considering that tumors in G1, GB, and GX may have a different biological behavior, they were excluded in this study. Ultimately, 551 samples containing information on 27,578 DNA methylation sites were included in this study, and the corresponding clinical information for each sample was also obtained from TCGA database. These 551 samples were separated into training dataset (first two thirds) and validation dataset (remaining one third) according to TCGA series number. The training dataset was applied for identifying and constructing prognostic biomarkers, and the validation dataset was used for verifying the accuracy of the biomarkers in predicting survival, thus determining the potential clinical predictive value.

### Statistical analyses

All statistical analyses were conducted using the R statistical package (R version 3.4.4) unless otherwise stated. OS was defined as the interval from the date of patient’s first diagnosis to the date of last known contact or death. The univariate Cox proportional hazard analysis was first performed in the training dataset to identify methylation markers significantly (*P* value cutoff < 0.05) correlated with patient survival as candidate markers. Then, the multivariate Cox regression analysis was carried out to further screen the factors associated with patient survival. In brief, two, three, four, five, and six genes were selected from the candidate markers exhaustively as covariates to construct models. Subsequently, AUC was used to measure and compare the model performance; the model with a higher predictive performance was eventually selected out. The model could be used to construct a risk score formula that would be helpful to predict survival. The prognostic risk scores for each patient were calculated based on this formula. According to their prognostic risk scores, these patients were ranked and further separated into “low-risk” and “high-risk” groups using the median risk score as the cutoff point. Patients with risk score higher than the median risk score were assigned to the high-risk group, whereas patients with lower risk were assigned to the low-risk group. After that, the Kaplan–Meier estimator, a nonparametric statistic, with log-rank test (Mantel–Cox) was used to calculate the cumulative survival time and compare the differences in OS between the two groups. Kaplan–Meier curves were drawn using the “survival” package. Finally, the ROC analysis was conducted with the “pROC” package using a categorical variable for OS ≤ 3 years compared with methylation biomarkers. AUC was calculated along with 95% confidence interval (CI). The larger the AUC is, the better the model is for the risk prediction [41]. And *Z* test was used to further compare the AUC of different biomarkers [42]. Additionally, the potential association between methylation and gene expression level was evaluated using the Spearman’s rank correlation test.

## Additional file

Additional file 1: Supplemental **Table S1-S2** and supplemental **Figure S1-S6**. (PDF 1215 kb)

## Abbreviations

AUC: Area under the ROC curve
CI: Confidence interval
OS: Overall survival
OSC: Ovarian serous cystadenocarcinoma
ROC: Receiver operating characteristic
TCGA: The Cancer Genome Atlas
FIGO: Federation Internationale des Gynaecologistes et Obstetristes
