# Supplementary material for: A five-DNA methylation signature act as a novel prognostic biomarker in patients with ovarian serous cystadenocarcinoma
Source: Clin Epigenetics. 2018 Nov 16;10:142. doi: 10.1186/s13148-018-0574-0 (PMC6240326; doi:10.1186/s13148-018-0574-0)
Supplement: Supplementary file 1 — Supplemental Table S1-S2 and supplemental Figure S1-S6. (PDF 1215 kb) [file 13148_2018_574_MOESM1_ESM.pdf]

Table S1 Five significantly survival-related methylation sites in training dataset.

Probe ID	Chromosomal location	Gene symbol	CGI coordinate	Feature type	*P* value[a]	Coef.[b]	*P* value[b]
cg05254747	chr8: 22366424 –22366425	*SLC39A14*	chr8: 22367006 –22367731	N_Shore	8.36E-03	–1.034	6.08E-03
cg13652336	chr8: 67951777 –67951778	*PREX2*	chr8: 67952190 –67952711	N_Shore	4.11E-02	2.433	4.60E-02
cg25123470	chr10: 101843965 –101843966	*KCNIP2*	chr10: 101843409 –101843669	S_Shore	1.54E-04	1.552	8.10E-05
cg06038133	chr17: 29617567 –29617568	*CORO6*	chr17: 29615514 –29618370	Island	3.63E-03	2.284	7.40E-05
cg04907664	chrX: 68829962 –68829963	*EFNB1*	chrX: 68828816 –68830409	Island	7.66E-03	–1.030	3.27E-03

[a]. in univariate Cox regression analysis;
[b]. in multivariate Cox regression analysis.

Table S2 The ROC results of five-DNA methylation signature and other known biomarkers.

**Signature**	**AUC**	**95% CI of AUC**	***P* value[a]**	**Type**	***P* value[b]**	**Ref**
Five-DNA methylation	0.715	0.62–0.81	< 0.001	Methylation		This study
Five-mRNA	0.671	0.54–0.80	0.014	Protein coding	0.294	[29]
Two-mRNA	0.556	0.42–0.69	0.424	Protein coding	0.025	[30]
Two-mRNA	0.507	0.37–0.65	0.917	Protein coding	0.005	[25]
HER2	0.504	0.37–0.64	0.954	Protein coding	0.005	[24]
CD44	0.605	0.47–0.74	0.132	Protein coding	0.088	[43]
MSX1	0.524	0.39–0.66	0.729	Protein coding	0.009	[15]
FGFRL1	0.544	0.41–0.68	0.524	Protein coding	0.018	[44]
SLC4A11	0.577	0.44–0.71	0.271	Protein coding	0.045	[45]
IGFBP7	0.577	0.44–0.71	0.266	Protein coding	0.045	[46]
Capn4	0.529	0.39–0.67	0.676	Protein coding	0.011	[47]
PXDN	0.627	0.49–0.76	0.068	Protein coding	0.140	[48]
TP73-AS1	0.519	0.38–0.66	0.781	lncRNA	0.008	[49]
HOTAIR	0.572	0.44–0.71	0.302	lncRNA	0.039	[27]
HOXD-AS1	0.556	0.42–0.69	0.302	lncRNA	0.025	[50]
BRCA1	0.615	0.51–0.72	0.036	Methylation	0.110	[26]
GATA4	0.606	0.50–0.71	0.052	Methylation	0.090	[51]
MYLK3	0.566	0.46–0.67	0.223	Methylation	0.014	[39]
FANCF	0.637	0.54–0.74	0.021	Methylation	0.170	[52]
HNF1B	0.546	0.44–0.65	0.401	Methylation	0.019	[51]
HOXA11	0.577	0.47–0.68	0.158	Methylation	0.045	[53]
HSPA1	0.581	0.48–0.69	0.138	Methylation	0.049	[54]
MLH1	0.604	0.50–0.71	0.056	Methylation	0.086	[55]
MSX1	0.588	0.48–0.69	0.107	Methylation	0.059	[15]
OPCML	0.575	0.47–0.68	0.167	Methylation	0.043	[5]

TMEM88	0.590	0.49–0.69	0.098	Methylation	0.062	[56]
TUSC3	0.555	0.45–0.66	0.314	Methylation	0.025	[57]
HOTAIR	0.542	0.44–0.65	0.437	Methylation	0.017	[28]

[a]. in ROC analysis;
[b]. in the statistical comparison (Z-test) between AUC value of corresponding signature and the five-DNA methylation signature.

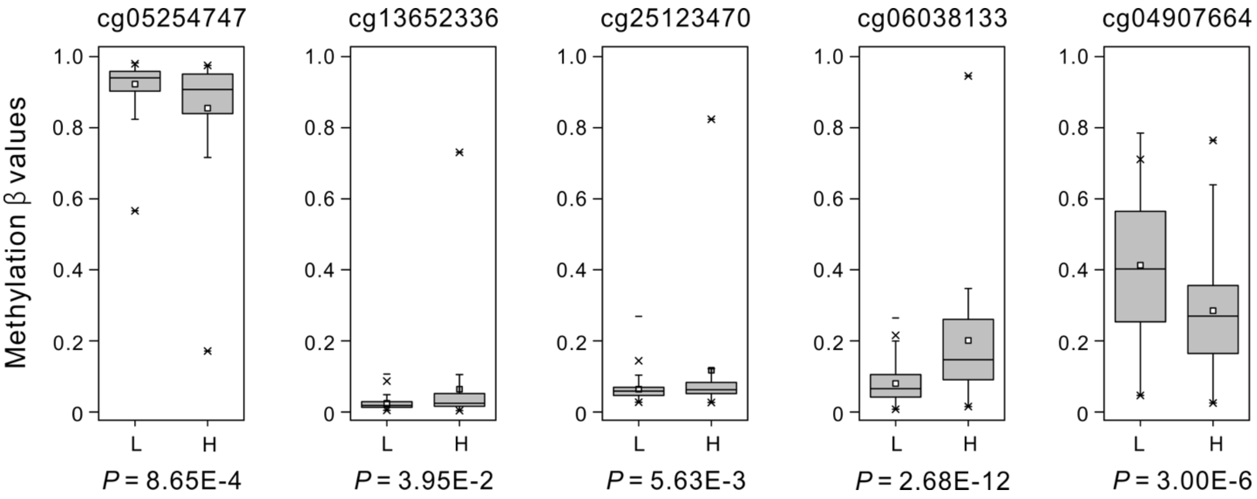

Figure S1. Boxplots of methylation β values in samples of patients in high-risk and low-risk groups in the validation dataset. "L" and "H" refer to the low-risk and high-risk group, respectively. Mann–Whitney *U* test was used to determine the differences between the two groups, and *P* values are shown below he graphs.

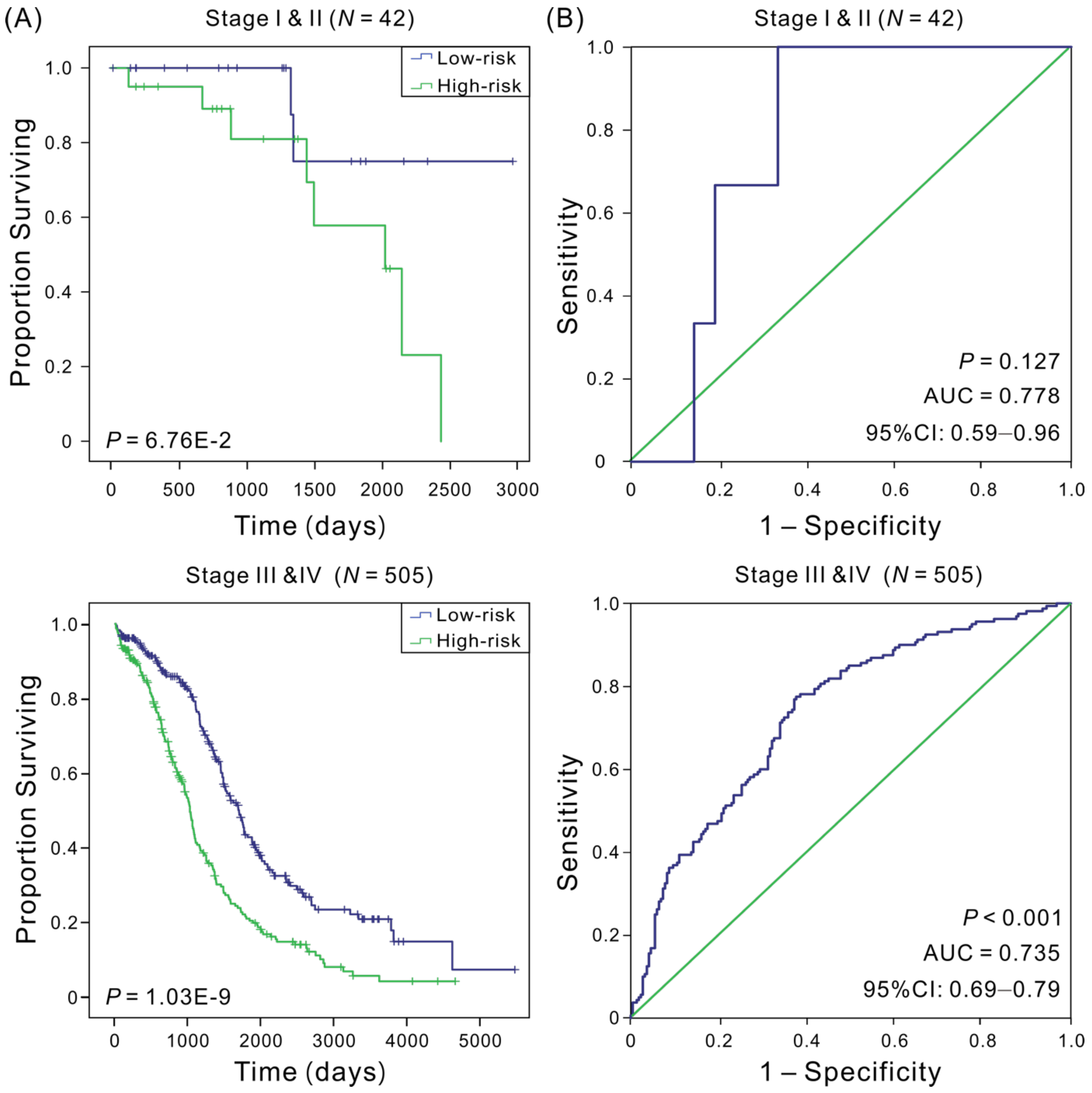

Figure S2. Kaplan–Meier and ROC analyses of OSC patients in early stage cohorts (stage I & II, *N* = 42) and advanced stage (stage III & IV, *N* = 505), respectively. **(A)** Kaplan–Meier estimates of the patients' OS for high-risk and low-risk patient in different stage cohorts, and the OS differences between two groups were determined by the two-sided log-rank test. It may be due to the limited number of samples, the signature could not significantly distinguish the high and low-risk patients in stage I & II; **(B)** ROC curves show the sensitivity and specificity of the five-DNA methylation signature in predicting the OS of patients.

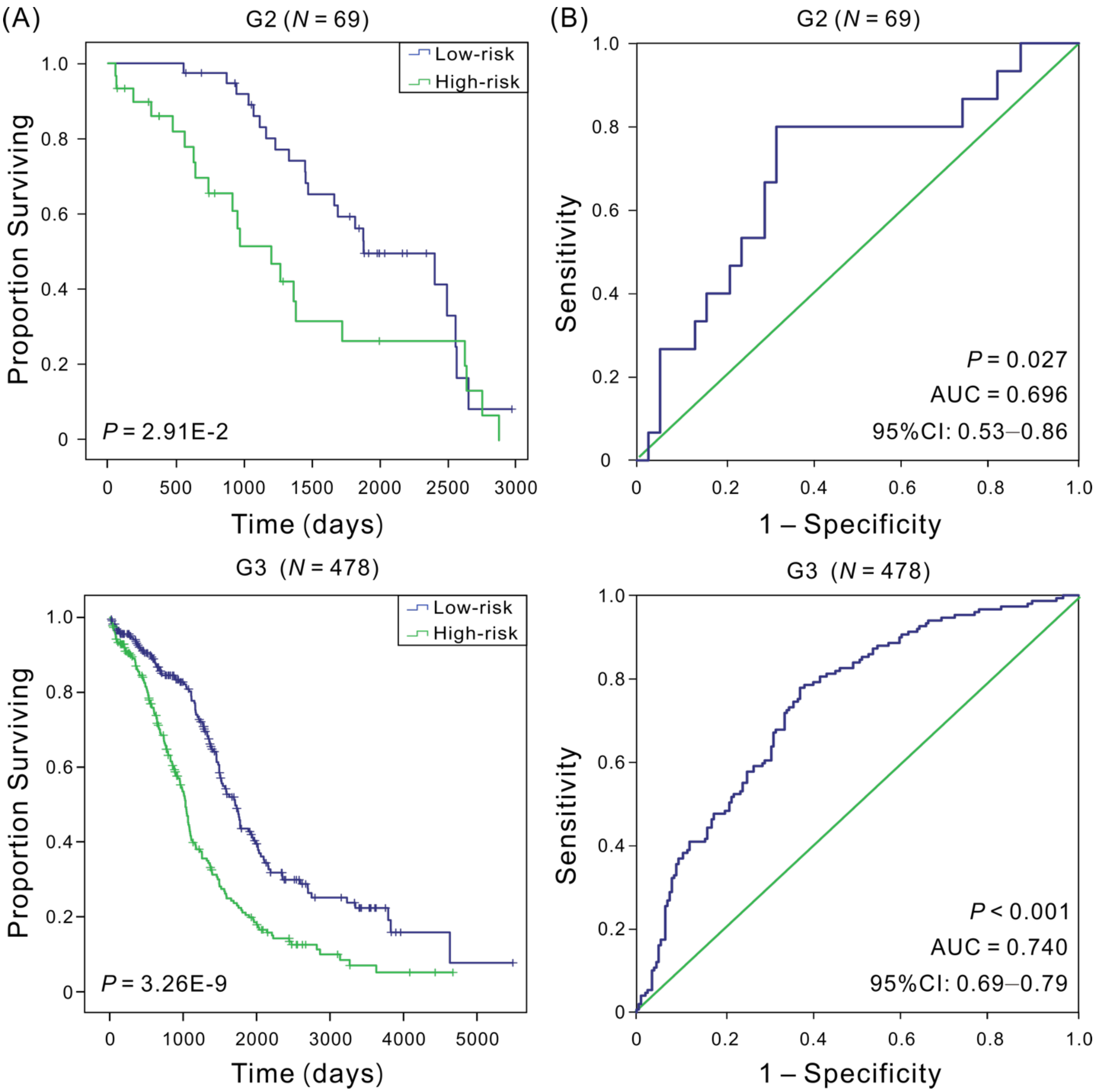

Figure S3. Kaplan-Meier and ROC analyses of OSC patients with Grade 2 (*N* = 69) and Grade 3 (*N* = 478), respectively. Kaplan–Meier estimates of the OS of patients **(A)** and ROC curves show the sensitivity and specificity of the five-DNA methylation signature in predicting the OS of patient **(B).**

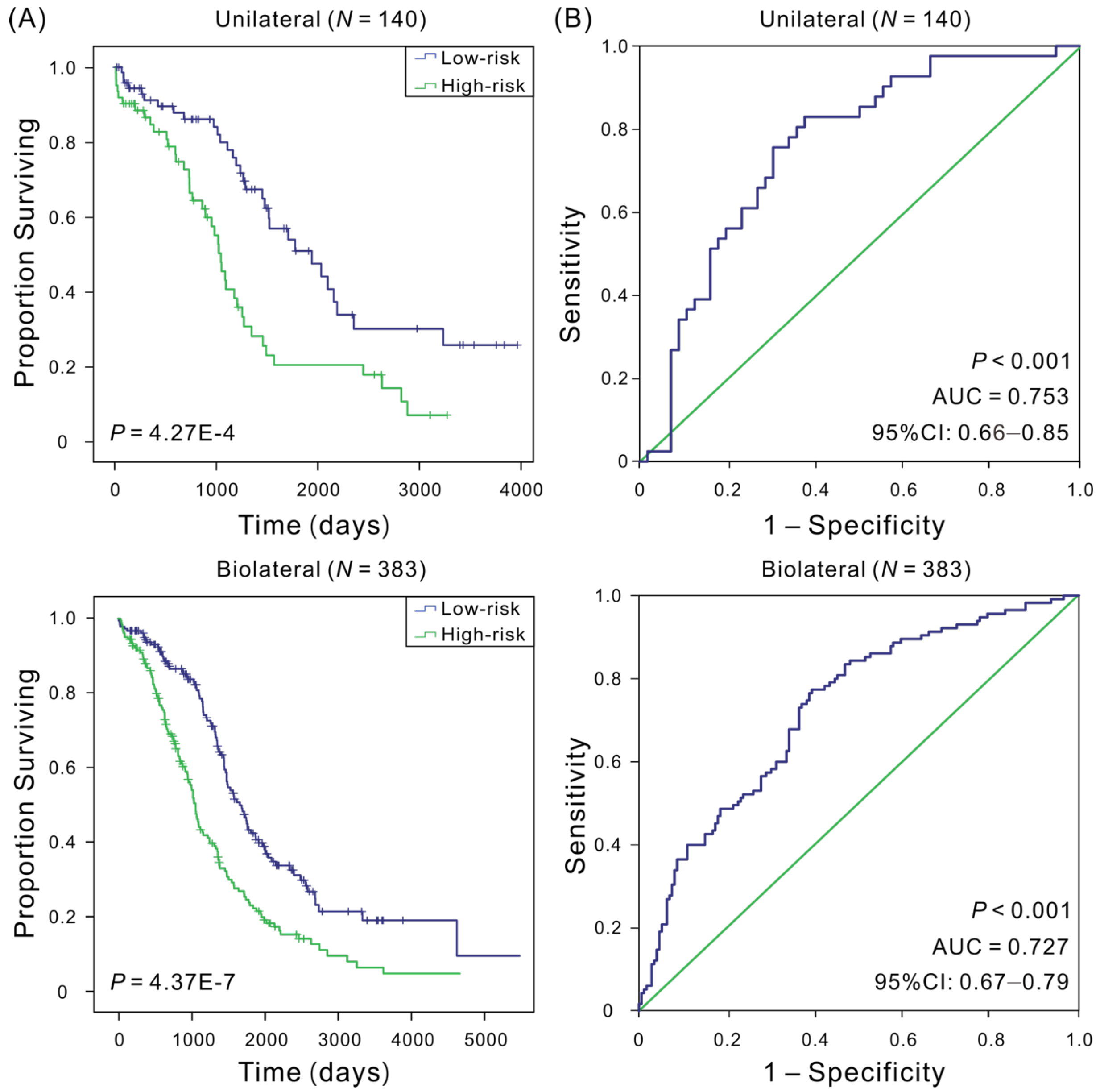

Figure S4. Kaplan–Meier (A) and ROC analyses (B) of OSC patients with samples the anatomic subdivision from unilateral (left or right, *N* = 140) and bilateral (*N* = 383), respectively.

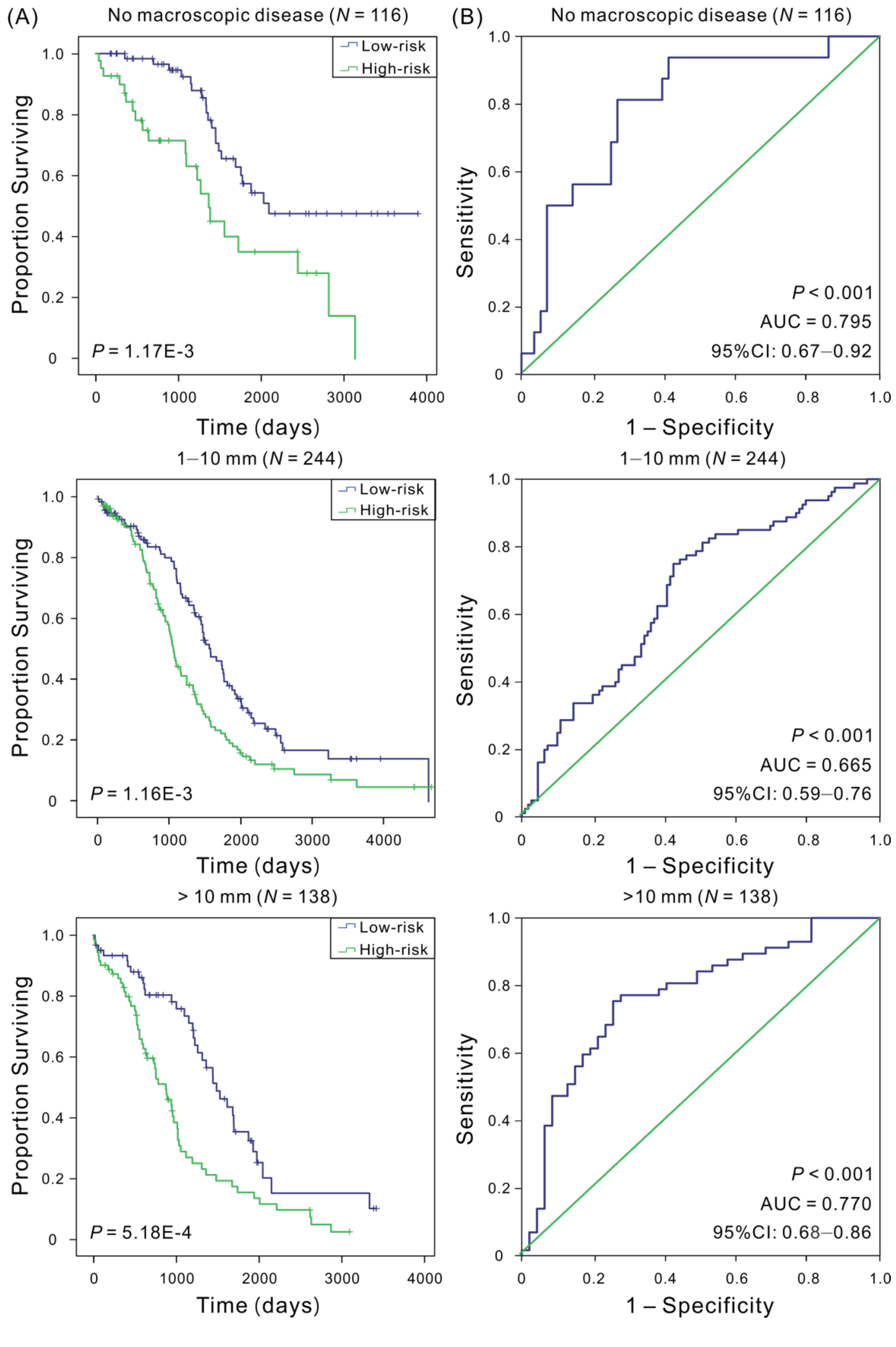

Figure S5. Kaplan–Meier (A) and ROC analyses (B) of OSC patients with different residual disease, including no macroscopic disease, 1–10 mm, and > 10 mm.

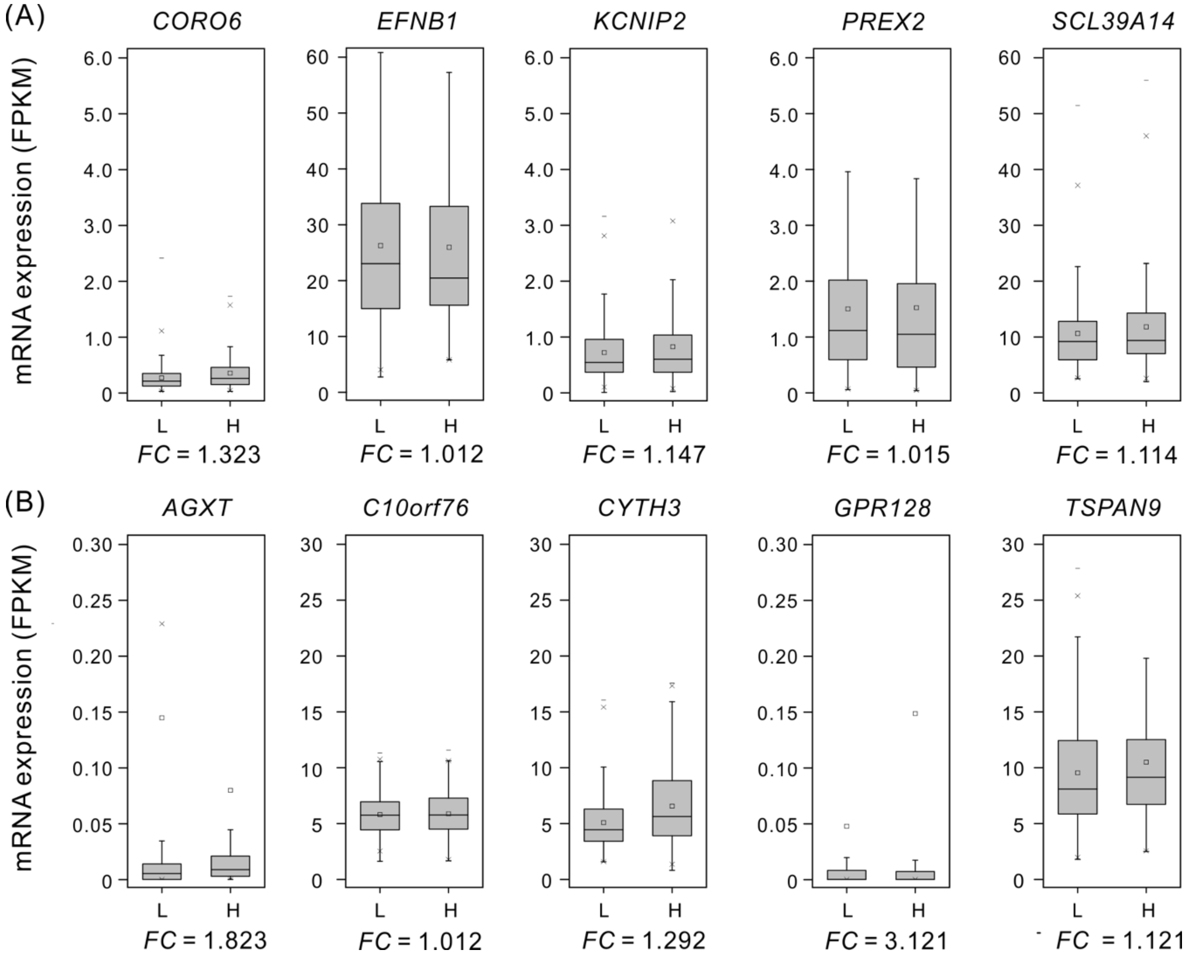

Figure S6. Boxplots of mRNA expression levels (FPKM) in patients in high-risk and low-risk groups. "L" and "H" refer to the low-risk and high-risk group, respectively. "*FC*" refers to the fold changes of expression levels. **(A)** Five genes that corresponds to the five DNA methylation sites in this study; (B) Five genes in the five-mRNA signature in our previous study.

Additional references

43. Gao Y, Foster R, Yang X, Feng Y, Shen JK, Mankin HJ, Hornicek FJ, Amiji MM, Duan Z, Up-regulation of CD44 in the development of metastasis, recurrence and drug resistance of ovarian cancer. Oncotarget 2015;6: 9313-26.

44. Tai H, Wu Z, Sun S, Zhang Z, Xu C, FGFRL1 Promotes Ovarian Cancer Progression by Crosstalk with Hedgehog Signaling. Journal of immunology research 2018;2018: 7438608.

45. Qin L, Li T, Liu Y, High SLC4A11 expression is an independent predictor for poor overall survival in grade 3/4 serous ovarian cancer. PloS one 2017;12: e0187385.

46. Gambaro K, Quinn MC, Caceres-Gorriti KY, Shapiro RS, Provencher D, Rahimi K, Mes-Masson AM, Tonin PN, Low levels of IGFBP7 expression in high-grade serous ovarian carcinoma is associated with patient outcome. BMC cancer 2015;15: 135.

47. Yang MF, Lou YL, Liu SS, Wang SS, Yin CH, Cheng XH, Huang OP, Capn4 overexpression indicates poor prognosis of ovarian cancer patients. Journal of Cancer 2018;9: 304-09.

48. Zheng YZ, Liang L, High expression of PXDN is associated with poor prognosis and promotes proliferation, invasion as well as migration in ovarian cancer. Annals of diagnostic pathology 2018;34: 161-65.

49. Li X, Wang X, Mao L, Zhao S, Wei H, LncRNA TP73AS1 predicts poor prognosis and promotes cell proliferation in ovarian cancer via cell cycle and apoptosis regulation. Molecular medicine reports 2018.

50. Zhang Y, Dun Y, Zhou S, Huang XH, LncRNA HOXD-AS1 promotes epithelial ovarian cancer cells proliferation and invasion by targeting miR-133a-3p and activating Wnt/beta-catenin signaling pathway. Biomedicine & pharmacotherapy = Biomedecine & pharmacotherapie 2017;96: 1216-21.

51. Bubancova I, Kovarikova H, Laco J, Ruszova E, Dvorak O, Palicka V, Chmelarova M, Next-Generation Sequencing Approach in Methylation Analysis of HNF1B and GATA4 Genes: Searching for Biomarkers in Ovarian Cancer. International journal of molecular sciences 2017;18.

52. Ding JJ, Wang G, Shi WX, Zhou HH, Zhao EF, Promoter Hypermethylation of FANCF and Susceptibility and Prognosis of Epithelial Ovarian Cancer. Reproductive sciences 2016;23: 24-30.

53. Fiegl H, Windbichler G, Mueller-Holzner E, Goebel G, Lechner M, Jacobs IJ, Widschwendter M, HOXA11 DNA methylation--a novel prognostic biomarker in ovarian cancer. International journal of cancer Journal international du cancer 2008;123: 725-9.

54. Jakobsson ME, Moen A, Davidson B, Falnes PO, Hsp70 (HSPA1) Lysine Methylation Status as a Potential Prognostic Factor in Metastatic High-Grade Serous Carcinoma. PloS one 2015;10: e0140168.

55. Gifford G, Paul J, Vasey PA, Kaye SB, Brown R, The acquisition of hMLH1 methylation in plasma DNA after chemotherapy predicts poor survival for ovarian cancer patients. Clinical cancer research : an official journal of the American Association for Cancer Research 2004;10: 4420-6.

56. de Leon M, Cardenas H, Vieth E, Emerson R, Segar M, Liu Y, Nephew K, Matei D, Transmembrane protein 88 (TMEM88) promoter hypomethylation is associated with platinum resistance in ovarian cancer. Gynecol Oncol 2016;142: 539-47.

57. Pils D, Horak P, Vanhara P, Anees M, Petz M, Alfanz A, Gugerell A, Wittinger M, Gleiss A, Auner V, Tong D, Zeillinger R, Braicu EI, Sehouli J, Krainer M, Methylation status of TUSC3 is a prognostic factor in ovarian cancer. Cancer 2013;119: 946-54.
